# Improving Awareness of Doctor Availability Within a Community Learning Disability Team

**DOI:** 10.1192/bjo.2025.10462

**Published:** 2025-06-20

**Authors:** Christopher Wilson

**Affiliations:** Leeds and York Partnership NHS Foundation Trust, Leeds, United Kingdom

## Abstract

**Aims:** To improve multidisciplinary team awareness of doctor availability within the East-North-East Leeds Community Learning Disability Team.

To create and implement a resource listing accurate availability per half-day, contact details, and named cover in cases of absence.

To improve team communication, reduce uncertainty, and streamline contact and escalation processes.

To utilise baseline and post-intervention outcome measures to assess improvements in practice.

**Methods:** This project was formulated following reports of uncertainty around doctor availability, inefficient methods of contact attempts, and with no current effective rota system in place.

A Plan-Do-Study-Act (PDSA) approach was taken to assess and adopt continuous improvement throughout.

Baseline questionnaires were distributed to staff to assess current levels of awareness of doctor availability, escalation pathways, and perceived usefulness of a planned resource.

Key stakeholders (consultants, resident doctors, managers, senior administrators) were engaged to adapt and expand a current limited senior doctor rota, to include all doctors, named cover, enhanced contact methods, and a wider audience. This utilised Outlook calendars, was displayed in the team office, and was updated weekly.

Post-intervention questionnaires were circulated to assess the impact of the intervention.

Further procedures were implemented to sustain this change, including commitments from permanent staff to take responsibility in maintaining the resource, standard operating procedures formulated for administrative and medical teams, and safeguards created to identify future issues encountered.

**Results:** Response rates from staff were 32% and 36% pre- and post-intervention respectively.

Staff confidence in knowing which doctors were available and when rose from 22% to 91%.

Staff knowledge of how to immediately contact an available doctor rose from 44% to 91%.

Staff knowledge of how to further escalate concerns rose from 67% to 91%.

91% of staff reported using the resource, 91% found it useful, and 82% found it accurate.

Further adaptations were made as the project progressed and in response to feedback and issues encountered.

**Conclusion:** This project successfully resulted in improving awareness of doctor availability in all domains measured, and was well received.

By developing a clear, accessible rota and engaging staff in its use, staff confidence and team communication improved.

This project led to lasting changes in practice, ensuring ongoing effectiveness sustained beyond the project’s conclusion.

Key points of discussion include engagement of key stakeholders in planning, implementing, and sustaining improvement, ensuring feasibility and longevity. Furthermore, reflecting on the effective use of PDSA principles with simple, measurable changes, and implementing ongoing review processes.

